# Disruption of Vitamin D Signaling Impairs Adaptation of Cerebrocortical Microcirculation to Carotid Artery Occlusion in Hyperandrogenic Female Mice

**DOI:** 10.3390/nu15183869

**Published:** 2023-09-05

**Authors:** Dorina Nagy, László Hricisák, Guillaume Peter Walford, Ágnes Lékai, Gábor Karácsony, Szabolcs Várbíró, Zoltán Ungvári, Zoltán Benyó, Éva Pál

**Affiliations:** 1 Institute of Translational Medicine, Semmelweis University, 1094 Budapest, Hungary; hricisak.laszlo@med.semmelweis-univ.hu (L.H.); walford.g@hotmail.fr (G.P.W.); agneslekai@gmail.com (Á.L.); kg.karacsony@gmail.com (G.K.); benyo.zoltan@med.semmelweis-univ.hu (Z.B.); 2 Cerebrovascular and Neurocognitive Disorders Research Group, Eötvös Loránd Research Network, Semmelweis University, 1094 Budapest, Hungary; 3 Department of Obstetrics and Gynecology, Semmelweis University, 1082 Budapest, Hungary; varbiro.szabolcs@med.semmelweis-univ.hu; 4 Department of Obstetrics and Gynecology, University of Szeged, 6725 Szeged, Hungary; 5 Workgroup for Science Management, Doctoral School, Semmelweis University, 1085 Budapest, Hungary; 6 Vascular Cognitive Impairment, Neurodegeneration and Healthy Brain Aging Program, Department of Neurosurgery, University of Oklahoma Health Sciences Center, Oklahoma City, OK 73104, USA; zoltan-ungvari@ouhsc.edu; 7 Department of Health Promotion Sciences, College of Public Health, University of Oklahoma Health Sciences Center, Oklahoma City, OK 73104, USA; 8 International Training Program in Geroscience, Doctoral School of Basic and Translational Medicine/Department of Public Health, Semmelweis University, 1089 Budapest, Hungary; 9 The Peggy and Charles Stephenson Cancer Center, University of Oklahoma Health Sciences Center, Oklahoma City, OK 73104, USA

**Keywords:** vitamin D signaling, cerebrovascular dysregulation, androgen excess, estrogen deficiency, carotid artery occlusion

## Abstract

Vitamin D deficiency contributes to the pathogenesis of age-related cerebrovascular diseases, including ischemic stroke. Sex hormonal status may also influence the prevalence of these disorders, indicated by a heightened vulnerability among postmenopausal and hyperandrogenic women. To investigate the potential interaction between sex steroids and disrupted vitamin D signaling in the cerebral microcirculation, we examined the cerebrovascular adaptation to unilateral carotid artery occlusion (CAO) in intact, ovariectomized, and hyperandrogenic female mice with normal or functionally inactive vitamin D receptor (VDR). We also analyzed the morphology of leptomeningeal anastomoses, which play a significant role in the compensation. Ablation of VDR by itself did not impact the cerebrocortical adaptation to CAO despite the reduced number of pial collaterals. While ovariectomy did not undermine compensatory mechanisms following CAO, androgen excess combined with VDR inactivity resulted in prolonged hypoperfusion in the cerebral cortex ipsilateral to the occlusion. These findings suggest that the cerebrovascular consequences of disrupted VDR signaling are less pronounced in females, providing a level of protection even after ovariectomy. Conversely, even short-term androgen excess with lacking VDR signaling may lead to unfavorable outcomes of ischemic stroke, highlighting the complex interplay between sex steroids and vitamin D in terms of cerebrovascular diseases.

## 1. Introduction

Cerebrovascular diseases and consequential vascular cognitive impairment represent a significant global burden, ranking among the leading causes of death and disability in older adults [1,2]. In addition to well-established risk factors such as hypertension [3], obesity [4,5,6], endocrine disorders [7,8,9,10], systemic atherosclerosis [11,12] and diabetes mellitus [13,14,15,16,17], growing evidence suggests that vitamin D deficiency (VDD) plays a crucial role as an emerging cardiovascular risk factor in age-related cerebrovascular pathologies [18,19,20,21]. The incidence of VDD has been on the rise in recent years, affecting a substantial proportion of the population, particularly in regions with limited sun exposure and inadequate dietary vitamin D intake [22,23,24,25,26]. Epidemiological studies indicate that VDD increases the risk of ischemic stroke development and poorer outcomes [27]. In recent years, the COVID-19 pandemic also highlighted the potential importance of maintaining optimal vitamin D levels for cerebrovascular well-being in high-risk patients [28,29,30], since evidence suggests that VDD may be associated with increased susceptibility to COVID-19 morbidity and COVID-19 itself has been linked to an elevated risk of cerebrovascular injury, stroke, and vascular cognitive impairment [31,32,33,34,35]. Understanding the interplay between vitamin D signaling and cerebrovascular health is of utmost importance for developing effective preventive and therapeutic strategies.

Our previous findings demonstrated the detrimental effects of VDD on cerebral vessels, characterized by adverse arterial remodeling, impaired vascular reactivity, and endothelial dysfunction [36,37,38]. These observations align with other studies highlighting the role of vitamin D in modulating vessel tone, regulating the release of endothelial vasoactive mediators, and exerting anti-inflammatory effects [39,40,41]. Importantly, we recently extended our understanding by investigating the severe functional consequences of disruption of vitamin D signaling on cerebrovascular adaptation to unilateral carotid artery occlusion (CAO) in male mice [42]. The CAO model serves as a valuable tool for unraveling crucial aspects of cerebrovascular regulation, providing insights into the complex interplay between vitamin D signaling and functional and structural cerebromicrovascular adaptation to insufficient blood supply of the brain.

While women generally exhibit lower incidence and mortality rates in cerebrovascular diseases compared to men, postmenopausal women and those in a hyperandrogenic state, including those with polycystic ovary syndrome (PCOS), are considered at-risk groups for stroke, especially in the presence of concomitant VDD [43,44,45,46]. Both vitamin D and sex steroids have significant effects on the cerebrovascular system [41,47]. Receptors and metabolic enzymes of sex steroids and vitamin D have been identified in the cerebral vasculature, enabling them to modulate cerebrovascular reactivity and regulate cerebral blood flow [39,47,48]. Sex steroids, particularly estrogen and androgens, exert significant influences on the regulation of cerebrovascular resistance and cerebral blood flow [49]. Estrogen, for example, regulates vasodilatory mechanisms through the modulation of endothelial factors such as nitric oxide (NO) and prostaglandins, while long-term exposure to testosterone appears to facilitate vasoconstriction [49,50]. Moreover, testosterone may promote vascular inflammation, contributing to the pathogenesis of vascular diseases, whereas estrogen suppresses it [47,51].

Given the recognized influence of sex steroids on cerebrovascular health and their potential interaction with VDD, we hypothesize that premenopausal healthy females may possess inherent protective mechanisms against the detrimental effects of VDD. However, we propose that the presence of androgen excess and estrogen deficiency in females could heighten the cerebrovascular consequences of VDD. Thus, our study aims to explore the efficacy of cerebrovascular adaptation to unilateral CAO in intact versus ovariectomized or hyperandrogenic female mice with functionally inactive VDR to disrupt vitamin D signaling. By investigating these interactions, we aim to gain insights into the complex interplay between sex steroids, vitamin D signaling, and cerebrovascular health in females.

## 2. Materials and Methods

### 2.1. Animals

The experiments were performed on adult female mutant mice carrying functionally inactive vitamin D receptors (VDR^∆/∆^), previously described [52], and their wild-type (WT) littermates with C57BL/6 genetic background, bred by intercrossing heterozygous animals. Mice were involved in the experiments at the age of 90–120 days. All animals received a rescue diet throughout their lives, enriched with calcium (2%), phosphorus (1.25%), and lactose (20%) (8852-S010, SM Rescue Diet VDR KO, ssniff Spezialdiäten GmbH, Soest, Germany) to ensure normalized calcium homeostasis [42,52]. The mice had ad libitum access to chow and water and were kept in a specific pathogen-free facility at constant temperature (19–22 °C) with a 12/12 light/dark cycle. All procedures were conducted according to the guidelines of Hungarian Law of Animal Protection (XXVIII/1998), and were approved by the National Scientific Ethical Committee on Animal Experimentation (PE/EA/00487-6/2021, approval date: 9 November 2021). Experiments were reported in compliance with the ARRIVE (Animal Research: Reporting in Vivo Experiments) guidelines.

### 2.2. Morphological Analysis of Leptomeningeal Collaterals

**Figure 1:**
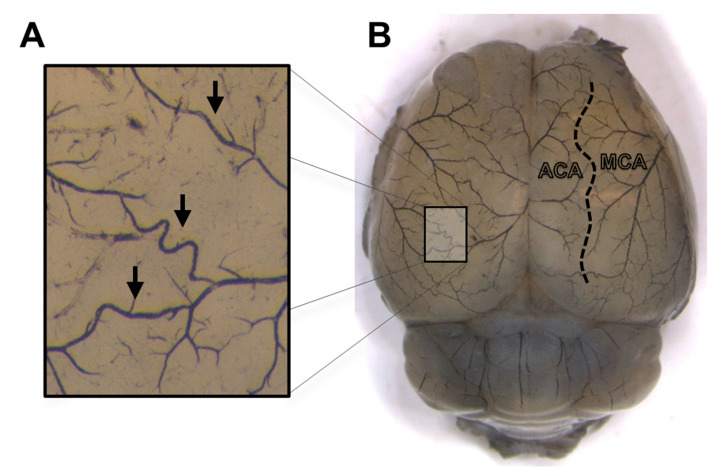
Representative image of a mouse brain with visualized vessels for the morphological analysis of leptomeningeal collaterals. (**A**) The arrows in the magnified image demonstrate collaterals between the branches of the middle cerebral artery (MCA) and the anterior cerebral artery (ACA), the number and tortuosity of which were analyzed. (**B**) The dashed line in the right hemisphere depicts the anastomotic line, which represents the border of the territories of the MCA and ACA.

For morphological analysis of leptomeningeal collaterals, the cerebrocortical vasculature was visualized in 5 VDR^∆/∆^ and 6 WT intact female mice (age: 90–120 days) by transcardial perfusion of heparinized saline solution (10 IU/mL) and a 6:1:6 proportioned mixture of black ink (Koh-I-Noor Hardmuth, Cescké Budejovice, Czech Republic), endorsing ink (Interaction-Connect, Gent, Belgium) and distilled water, as previously described [42]. The brains were removed after decapitation and fixed with 4% formaldehyde solution. The morphology of leptomeningeal collaterals connecting the branches of the anterior cerebral artery (ACA) and the middle cerebral artery (MCA) was evaluated on digital pictures taken with a digital camera attached to a microscope (Leica MC 190 HD and Leica M80, Leica Microsystems, Wetzlar, Germany) (Figure 1). The number of collaterals, tortuosity index (the ratio of vessel curve length over the line distance between the two ends of the vessel), and the distance between the anastomotic line (a line connecting the half-distance points between the nearest branching points of the ACA and MCA branches) and the midline was determined using ImageJ software (ImageJ 1.5 NIH, Bethesda, MD, USA) [42].

### 2.3. Ovariectomy and Testosterone Treatment

At three months of age, mice were selected for either surgical ovariectomy or transdermal testosterone treatment. 10 VDR^∆/∆^ and 10 WT female mice were subjected to bilateral ovariectomy (OVX), performed under isoflurane (2%) anesthesia and sterile conditions (OVX-VDR^∆/∆^ and OVX-WT groups). After the surgery, the mice received ceftriaxone (100 μg/g body weight, i.p., Ceftriaxon Kabi; Fresenius Kabi Deutschland GmbH, Bad Homburg, Germany) as a prophylactic antibiotic and acetaminophen (200 μg/g body weight, i.p., Paracetamol Kabi; Fresenius Kabi Hungary, Budapest, Hungary) as an analgesic treatment. Their health status was checked every day until the experiments, which were conducted five weeks after OVX. 10 VDR^∆/∆^, and 10 WT female mice received daily transdermal testosterone treatment (33 μg/g body weight, Androgel 1%, Laboratories Besins International S.A., Paris, France) for five weeks (TT-VDR^∆/∆^ and TT-WT groups) [38]. The fur was removed on a small surface of the back with a mouse razor under isoflurane (2%) anesthesia. This procedure was repeated when the fur had grown back (once or twice a week, if necessary). The analytically measured amount of Androgel 1% was applied to the skin every day at the same time to minimize blood level fluctuations. Each mouse’s condition was checked daily, and no skin irritation was observed. The ovariectomized, and testosterone-treated mice were single-housed for five weeks to ensure a safe recovery from the surgery and to avoid fighting injuries. Body weight was measured before OVX/testosterone treatment and five weeks after (at the time of the in vivo cerebrocortical blood flow measurements) to determine weight gain. Then, 10 VDR^∆/∆^ and 10 WT intact mice (age: 90–120 days) were assigned to control groups. Table 1 summarizes the experimental design in the in vivo cerebrocortical blood flow measurements.

### 2.4. Vaginal Cytology

**Figure 2:**
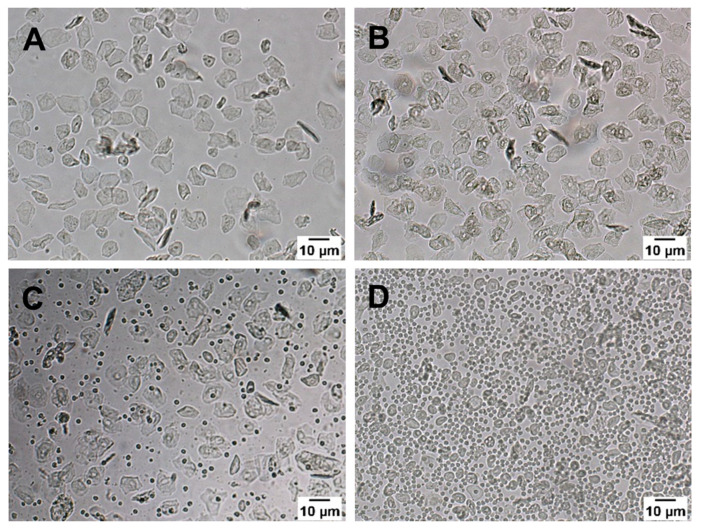
Four phases of the estrus cycle observed under light microscopy in unstained samples: (**A**) proestrus with a large proportion of nucleated epithelial cells and less cornified epithelial cells; (**B**) estrus with a large proportion of cornified epithelial cells and less nucleated epithelial cells; (**C**) metestrus with leucocytes, nucleated and cornified epithelial cells; and (**D**) diestrus with leucocytes and a few nucleated epithelial cells. Intact and testosterone-treated mice were selected for the in vivo experiments in the diestrus phase, which can be accurately identified on account of the high proportion of leucocytes.

Vaginal cytology was examined in intact, ovariectomized, and testosterone-treated VDR^∆/∆^ and WT female mice for at least five consecutive days before performing in vivo cerebrocortical blood flow measurements. To identify the animals’ stage of the estrus cycle, vaginal smears were collected from awake animals in the early mornings by gently flushing the vaginal canal with 0.1 mL saline solution using syringes with blunt needles. The estrus cycle was determined by evaluating the proportion of leucocytes, cornified epithelial cells, and nucleated epithelial cells in unstained samples under light microscopy (Zeiss Axio Imager.A1, Göttingen, Germany) [53]. Figure 2 shows the four phases of the estrus cycle. In the ovariectomized groups, the successful removal of the ovaries was validated by a suppressed estrus cycle [54], according to daily smear tests conducted for at least five consecutive days. The effect of testosterone treatment on the estrus cycle was examined, respectively. To avoid hormonal biases, intact control, and testosterone-treated mice were selected for the in vivo experiments in the diestrus phase (Figure 2D).

### 2.5. Determination of Cerebrocortical Blood Flow Changes after Carotid Artery Occlusion

#### 2.5.1. Surgical Procedures

The in vivo cerebrocortical blood flow measurements were performed five weeks after ovariectomy or after the testosterone treatment. Intact females served as control groups. First, the left femoral artery was cannulated under isoflurane (2%) anesthesia to measure the blood pressure changes during Laser-speckle imaging [42,55]. After this procedure, ketamine (100 μg/g body weight Calypsol; Gedeon Richter, Budapest, Hungary)-xylazine (10 μg/g body weight, CP-Xylazine; CP-Pharma, Burgdorf, Germany) was applied intraperitoneally as an anesthetic. An intratracheal cannula was inserted to ensure free breathing. The left common carotid artery was carefully separated from the surrounding tissues, and a loose knot was placed around it for later occlusion. Plantar nociception was frequently checked to maintain a sufficient level of anesthesia, and if necessary, ketamine-xylazine was administered again. All surgical procedures were performed on a heating pad (body temperature: 37–38 °C) controlled by a rectal thermometer under a stereomicroscope (Wild M3Z, Heerbrugg, Switzerland) [42,55].

#### 2.5.2. Measurement of Cerebrocortical Blood Flow, Arterial Blood Pressure, and Blood Gas Parameters

**Figure 3:**
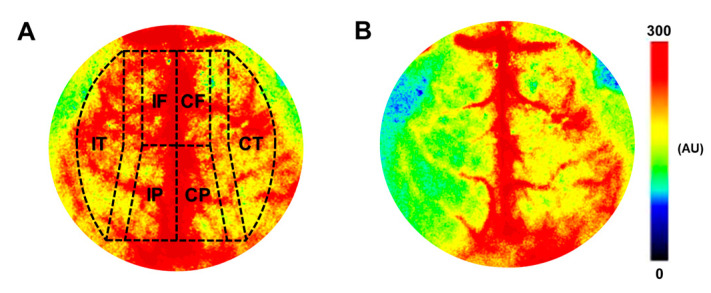
Cerebrocortical blood flow (CoBF) measurement with Laser-speckle imaging. (**A**) Representative image of baseline CoBF before unilateral carotid artery occlusion, indicating the localization of the regions of interest in the cerebral cortex, where the CoBF changes were examined separately. Large vessels (outlined in red color) were excluded from evaluation to analyze the microcirculation exclusively. (**B**) Representative image indicating hypoperfusion after carotid artery occlusion in the ipsilateral hemisphere. Blue-green color illustrates hypoperfusion, while red represents higher blood flow. AU: arbitrary units; IF: frontal region of the ipsilateral hemisphere; IP: parietal region of the ipsilateral hemisphere; IT: temporal region of the ipsilateral hemisphere; CF: frontal region of the contralateral hemisphere; CP: parietal region of the contralateral hemisphere; CT: temporal region of the contralateral hemisphere.

In vivo Laser-speckle imaging (PeriCam PSI; Perimed, Järfälla, Stockholm, Sweden) was used to measure cerebrocortical blood flow (CoBF) changes triggered by CAO [42,55]. After the surgery, the mouse was placed in a stereotaxic head holder, and the skull was exposed by a midline incision on the scalp to allow the laser-light to reach the cortex. The systemic mean arterial blood pressure (MABP) was continuously measured using the femoral artery cannula (MP100 System and AcqKnowledge 3.72 Software, Biopac Systems Inc., Goleta, CA, USA). A pulse oximeter (MouseOx Plus, Starr Life Sciences Corp., Oakmont, PA, USA) was placed on the right hind limb to monitor oxygen saturation, heart rate, and respiratory rate. In order to reverse the α-2-agonist effects of xylazine, atipamezole (1 μg/g ip.; Sigma-Aldrich, St. Louise, MO, USA), an α-2-antagonist agent, was applied [55]. After the blood pressure had stabilized and the depth of anesthesia was sufficient, baseline CoBF and MABP were recorded. Then, the occlusion was carried out by tightening the loose knot around the left carotid artery, and the CoBF changes were recorded for five minutes. Two phases of the adaptation were examined separately: 0–30 s was considered the acute phase, and 31–300 s the subacute phase. The CoBF changes were expressed as a percentage of the baseline reference value (100%), calculated as the average baseline CoBF based on the one-minute recording before CAO. The CoBF changes were measured in three cerebrocortical regions in both hemispheres: frontal, parietal, and temporal cortices (Figure 3) [55]. To quantify the CoBF reductions, the area over the curve (AOC) was calculated for each animal in both phases (i.e., acute, subacute) and all regions. Arterial blood gas tensions (pCO_2_, pO_2_), acid–base parameters (pH, bicarbonate concentration), O_2_ saturation, and plasma ion concentrations (Ca^2^⁺, Na⁺, K⁺, Cl^−^) were determined at the end of each measurement by radiometric analysis (ABL80 FLEX Blood Gas Analyzer, Radiometer, Brønshøj, Denmark) following arterial blood sampling using the femoral artery cannula. The experiment was not evaluated if arterial O_2_ saturation was lower than 90%, CO_2_ tension was outside 25–55 mmHg, or systemic MABP was outside 70–120 mmHg. The complete occlusion of the carotid artery was verified under a stereomicroscope [42,55]. Because of abnormal arterial blood gas tensions or low MABP values, 1-1 mouse was excluded from OVX-WT and TT-VDR^∆/∆^ groups, whereas two mice from VDR^∆/∆^ group. After the experiments, body weight, heart weight, brain weight, and tibial length were also measured.

### 2.6. Measurement of Testosterone Levels

Arterial blood samples were taken using the femoral artery cannula after each experiment for later serum testosterone level measurements. Testosterone was measured in serum samples using ultra-high-performance liquid chromatography (UHPLC)–tandem mass spectrometry (MS/MS) with a method described previously, with slight modification [56]. Briefly, 200 µL serum was deproteinized using acetonitrile containing internal standards, followed by the solid-phase extraction of the analytes on a Phenomenex Strata-X 60 mg cartridge (Gen-Lab Ltd., Budapest, Hungary). The eluates were evaporated to dryness and reconstituted in methanol–water 1:1. The quantitative analysis was accomplished on a Shimadzu Nexera X2 UHPLC coupled to an LCMS-8060 triple quadrupole mass spectrometer using reversed-phase liquid chromatographic separation on a Phenomenex Kinetex XB-C18 50 × 2.1 mm (particle size: 1.7 µm) and a Phenomenex Kinetex Biphenyl 50 × 2.1 mm (particle size: 1.7 µm) analytical column, connected in series, and gradient elution with a mixture of water and methanol, both containing 0.3% formic acid. Multiple reaction monitoring was employed for mass selective detection. Calibration was performed by employing six-point calibration curves and applying 1/concentration^2^ weights. Chromsystems 6PLUS1 Multilevel Serum Calibrator Panel 1 and Panel 2 lyophilized serum calibrator sets and MassCheck Steroid Panel 1 and 2 serum controls were processed along with the experimental samples (ABL&E-Jasco Hungary Ltd., Budapest, Hungary). Estrogen levels were not measured by reason of insufficient volume of plasma samples.

### 2.7. Statistical Analysis

Normal distribution of datasets was confirmed by the Shapiro–Wilk test, and data are presented as mean ± SEM. When the data distribution was not normal, data are presented as median and interquartile range. In the morphological analysis, *p*-values were calculated using Student’s unpaired t-test. The significance of weight gain was determined by Student’s paired t-test or Wilcoxon test. The statistical significance of the results obtained from the in vivo experiments was assessed by two-way ANOVA followed by Tukey’s post hoc test. If the data distribution was not normal, ANOVA was carried out after data transformation. Statistical analysis was performed using GraphPad Prism software (v.8.0, GraphPad Software Inc., La Jolla, CA, USA), and *p* < 0.05 was considered a statistically significant difference.

## 3. Results

### 3.1. Anatomical and Physiological Parameters

#### 3.1.1. Anatomical Traits

To examine the effect of disrupted vitamin D signaling and alterations of sex steroid levels on general anatomical parameters in female mice, body weight, heart weight, brain weight, and tibial length were measured. Functional inactivation of VDR signaling alone did not cause any difference in these parameters in intact females, although the tibial length of VDR^∆/∆^ mice was shorter than that of OVX-WT and TT-WT mice (Table 2). Interestingly, testosterone-treated WT mice had significantly higher body weight compared to intact, ovariectomized, and testosterone-treated VDR^∆/∆^ mice as well as intact WT mice (Table 2). The five-week testosterone treatment caused significant weight gain in both TT-WT (before treatment: 21.39 (20.72–28.40) g, after treatment: 23.85 (21.55–30.77) g; Wilcoxon test, * *p* < 0.05, *n* = 10) and TT-VDR^∆/∆^ (before treatment: 20.45 ± 0.85 g, after treatment: 22.22 ± 0.82 g; paired T-test, *** *p* < 0.001, *n* = 10) groups. Similarly, ovariectomy resulted in a significant increase in body weight after five weeks in both OVX-WT (before treatment: 21.86 ± 0.36 g, after treatment: 23.56 ± 0.42 g; paired *t*-test, **** *p* < 0.0001, *n* = 10) and OVX-VDR^∆/∆^ (before treatment: 19.57 ± 0.51 g, after treatment: 21.82 ± 0.30 g; paired *t*-test, ** *p* < 0.01, *n* = 10) groups.

#### 3.1.2. Morphology of Leptomeningeal Collaterals in Intact Females

**Figure 4:**
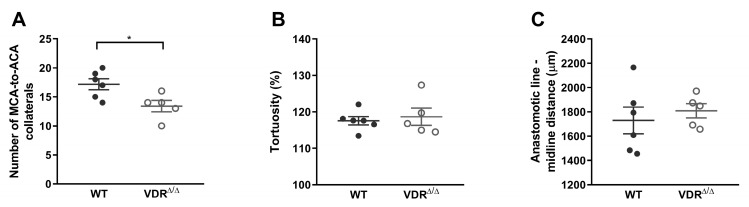
Evaluation of the morphology of leptomeningeal collaterals in intact WT and VDR^∆/∆^ female mice. The number of MCA-to-ACA collaterals was significantly lower in VDR^∆/∆^ females compared to WT mice (**A**). No significant difference was found in the tortuosity of collaterals between the experimental groups (**B**). The localization of the anastomotic line was not influenced by the lack of VDR signaling as the anastomotic line-midline distance in VDR^∆/∆^ females did not differ from WT mice (**C**). Data are presented as mean ± SEM, *n*_WT_ = 6, *n*_VDR_^∆/∆^ = 5, where *n* refers to the number of brains analyzed. Student’s unpaired test (* *p* < 0.05) was used for statistical analysis. MCA: middle cerebral artery; ACA: anterior cerebral artery.

The morphology and number of leptomeningeal collaterals can exert a considerable influence on the outcome of an ischemic stroke [57]. In case of a primary artery blockade, they can provide an alternative way for the blood flow to the unsupplied territory [58]. In a previous study, we discovered morphological alterations of leptomeningeal anastomoses in male VDR^∆/∆^ mutant mice [42]. Considering the potential gender differences in VDD [37,59], we hypothesized that females might be more protected from unfavorable morphological changes. To test this hypothesis, we examined the number of collaterals between the branches of MCA and ACA, and their tortuosity, and determined the anastomotic line’s distance from the midline to differentiate between the cortical territories supplied by the MCA and ACA [42]. In VDR^∆/∆^ females, the number of MCA-to-ACA collaterals significantly reduced compared to control mice (Figure 4A). However, the tortuosity (Figure 4B) and localization of the vessels (Figure 4C) did not differ among the experimental groups. These findings imply less adverse consequences of disrupted vitamin D signaling on collateral development in females compared to our previous findings in males, where both the increased tortuosity and the shifted anastomotic line indicated more severe alterations [42].

#### 3.1.3. Validation of Testosterone Treatment and Ovariectomy

Testosterone levels were measured by UHPLC in serum samples taken after the in vivo experiments using the femoral artery cannula. Although testosterone levels in intact control and ovariectomized mice were below the detection limit (0.05 ng/mL), both were elevated in the testosterone-treated groups, which verifies the successfulness of the transdermal treatment. The testosterone levels measured in TT-WT (1.51 ± 0.25 ng/mL) and TT-VDR^∆/∆^ (1.06 ± 0.18 ng/mL) did not differ significantly from each other (*n* = 8-8, unpaired t-test). To examine the impact of ovariectomy on estrus cyclicity, the estrus cycle of females was determined for five consecutive days before the in vivo experiments. Intact females had normal estrus cycles, while the ovariectomized groups demonstrated acyclicity five weeks after the surgery indicating the successful removal of the ovaries.

#### 3.1.4. In Vivo Blood Pressure Measurement

The systemic arterial blood pressure was continuously monitored during the in vivo cerebrocortical blood flow measurements. Baseline MABP was defined as the one-minute-long recording before CAO, which was conducted at time point zero. MABP was monitored for five more minutes after CAO. Average MAPB values were calculated for each minute for all mice and then compared to each other (Table 3). None of the experimental groups’ blood pressure differed significantly from the others. All groups’ blood pressure was in the physiological range and within the cerebral autoregulation range. Therefore, we can dismiss the possibility that blood pressure differences between the groups resulted in cerebrocortical blood flow alterations. Carotid artery occlusion induced only a slight increase in blood pressure (Table 3).

#### 3.1.5. Analysis of Blood Gas, Acid–Base Parameters, and Plasma Ion Concentrations

At the end of the in vivo experiments, arterial blood gas (pCO_2_, pO_2_), acid–base parameters (pH, bicarbonate concentration), hematocrit level, plasma ion concentrations (Na⁺, K⁺, Ca^2^⁺, Cl^−^), and O_2_ saturation were measured in arterial blood samples. None of these parameters exhibited differences among the experimental groups (Table 4). Similar calcium ion concentrations can prove the efficacy of the rescue diet in normalizing calcium homeostasis in VDR-mutant mice [52]. As arterial blood gas tensions were within the physiological range, we can exclude the possibility that they altered the cerebrovascular autoregulatory capacity in any of the experimental groups (Table 4).

### 3.2. Regional Cerebrocortical Blood Flow Changes after Carotid Artery Occlusion

**Figure 5:**
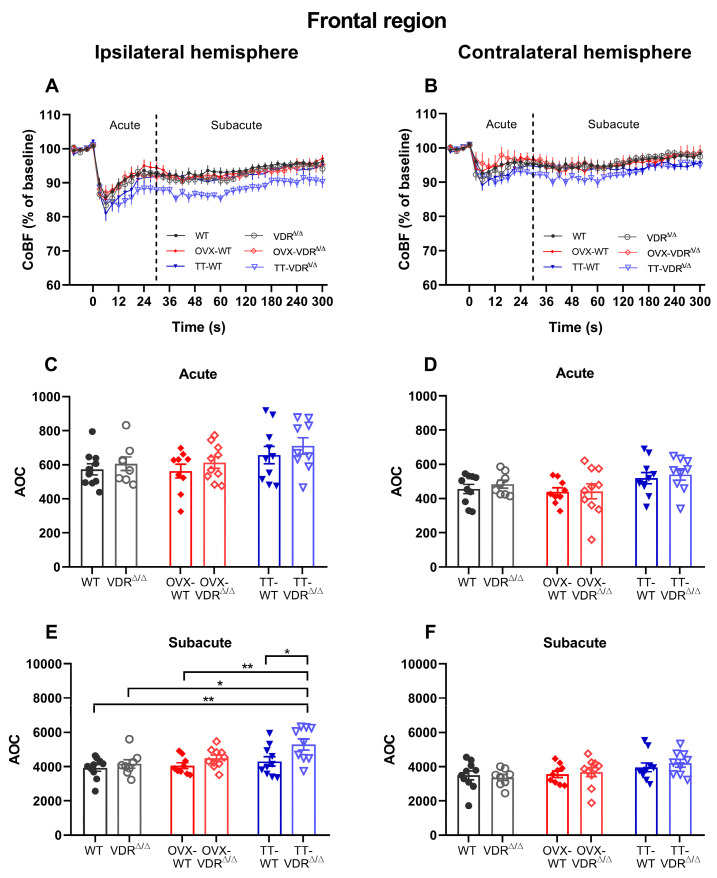
Cerebrocortical blood flow (CoBF) changes after carotid artery occlusion (CAO) in the frontal cortex of the ipsilateral (**A**) and contralateral (**B**) hemispheres. The zero point on the time scale represents the moment of CAO, and the dashed line splits the acute (0–30 s) and subacute (31–300 s) phases of adaptation (**A**,**B**). The CoBF reductions were quantified as the areas over the curve (AOCs) for each mouse and analyzed separately in the acute (**C**,**D**) and subacute (**E**,**F**) phases of adaptation. In the acute phase, no differences were observed among the experimental groups in any of the hemispheres (**C**,**D**). However, in the subacute phase, TT-VDR^∆/∆^ mice had significantly higher AOC values compared to intact females (VDR^∆/∆^, WT) and OVX-WT mice, which indicates a prolonged blood flow reduction in the TT-VDR^∆/∆^ group (**E**). In the subacute phase of the contralateral hemisphere, no significant differences were observed among the experimental groups (**F**). Data are presented as mean ± SEM, n_WT_ = 10, n_VDR_^∆/∆^ = 8, n_OVX-WT_ = 9, n_OVX-VDR_^∆/∆^ = 10, n_TT-WT_ = 10, n_TT-VDR_^∆/∆^ = 9. Two-way ANOVA followed by Tukey’s post hoc test (* *p* < 0.05, ** *p* < 0.01) was used for statistical analysis.

The CoBF changes after CAO were analyzed in three cerebrocortical regions (frontal, parietal, temporal) in both hemispheres, respectively (Figure 3). At the moment of the occlusion, the blood flow abruptly decreases in the ipsilateral hemisphere, but after a short time, the blood flow starts to approach the baseline level due to the activation of compensatory mechanisms [55]. Conversely, in the contralateral hemisphere, a negligible disturbance of blood flow is expected due to the presence of the intact carotid artery. In order to quantitatively assess the CoBF reductions after CAO and compare them among all experimental groups, the areas over the curve (AOCs) were calculated for each mouse separately in the acute (0–30 s) and subacute (31–300 s) phases of adaptation. The frontal region is supplied predominantly by the azygous anterior cerebral artery (AACA), which is supplied from both sides of the circle of Willis [55]. Figure 5A,B represent the CoBF changes after CAO in the frontal region of each hemisphere, from which the AOC values were calculated (Figure 5C–F). In the ipsilateral hemisphere, no significant difference was discovered between the groups in the acute phase (Figure 5C), which indicates a similar extent of hypoperfusion in the groups. However, a considerable difference was observed in the subacute phase: based on the higher AOC values of the TT-VDR^∆/∆^ group compared to intact females (i.e., VDR^∆/∆^, WT) and OVX-WT mice, TT-VDR^∆/∆^ mice suffered a significantly prolonged blood flow reduction compared to these groups (Figure 5E). The contralateral hemisphere was less impacted, as no significant differences were found in the calculated AOCs in either the acute (Figure 5D) or in the subacute (Figure 5F) phases of adaptation.

**Figure 6:**
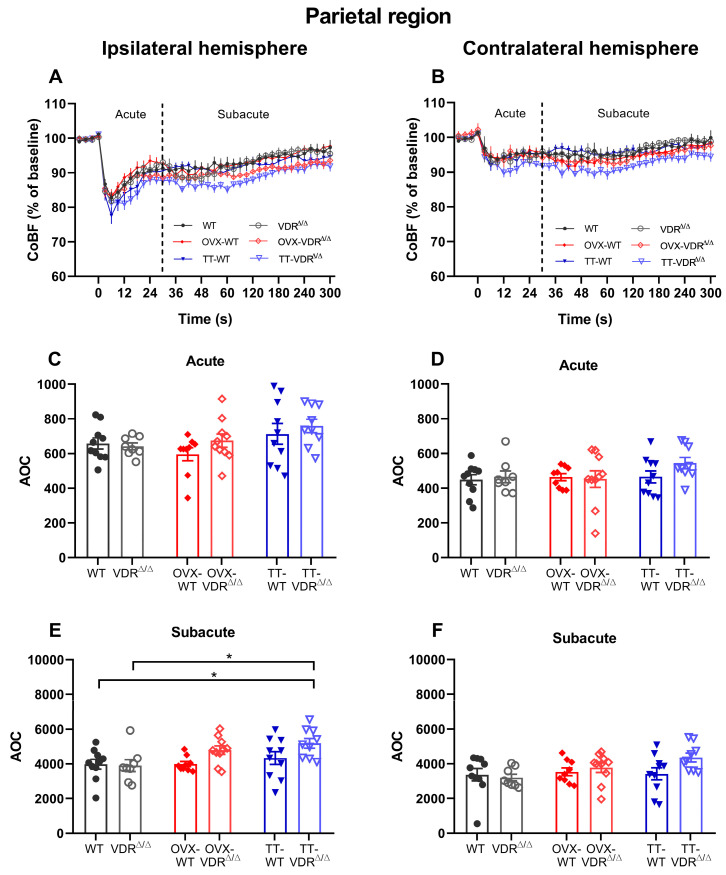
Cerebrocortical blood flow (CoBF) changes after carotid artery occlusion (CAO) in the parietal cortex of the ipsilateral (**A**) and contralateral (**B**) hemispheres. The zero point on the time scale represents the moment of CAO, and the dashed line splits the acute (0–30 s) and subacute (31–300 s) phases of the adaptation (**A**,**B**). The CoBF reductions were quantified as the areas over the curve (AOCs) for each mouse and analyzed separately in the acute (**C**,**D**) and subacute (**E**,**F**) phases of adaptation. In the acute phase, no differences were found among the experimental groups in any of the hemispheres (**C**,**D**). However, the increased AOC of TT-VDR^∆/∆^ mice in the ipsilateral hemisphere implies a delayed recovery during the subacute phase compared to the intact females (WT, VDR^∆/∆^) (**E**). In the subacute phase of the contralateral hemisphere, no significant differences were observed among the experimental groups (**F**). Data are presented as mean ± SEM, n_WT_ = 10, n_VDR_^∆/∆^ = 8, n_OVX-WT_ = 9, n_OVX-VDR_^∆/∆^ = 10, n_TT-WT_ = 10, n_TT-VDR_^∆/∆^ = 9. Two-way ANOVA followed by Tukey’s post hoc test (* *p* < 0.05) was used for statistical analysis.

The blood resources of the parietal region are mainly given by the AACA, but additional supply can also be obtained from the posterior cerebral artery [55]. The CoBF curves are represented in Figure 6A (ipsilateral hemisphere) and Figure 6B (contralateral hemisphere). According to the calculated AOC values, no differences were found among the groups in the acute phase of this ipsilateral region (Figure 6C). Likewise, in the frontal region, TT-VDR^∆/∆^ mice had higher AOC values in the subacute phase in the parietal region of the ipsilateral hemisphere compared to both WT and VDR^∆/∆^ animals (Figure 6E), indicating a delayed recovery. In the contralateral hemisphere, no significant differences were observed among the groups in either the acute (Figure 6D) or in the subacute (Figure 6F) phases.

**Figure 7:**
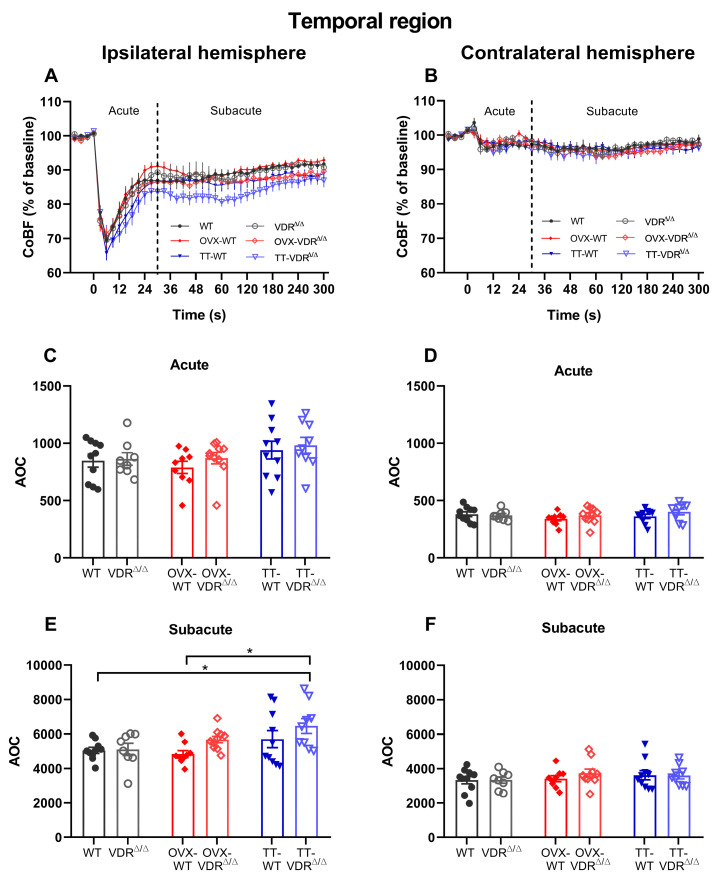
Cerebrocortical blood flow (CoBF) changes after carotid artery occlusion (CAO) in the temporal cortex of the ipsilateral (**A**) and contralateral (**B**) hemispheres. The zero point on the time scale represents the moment of CAO, and the dashed line splits the acute (0–30 s) and subacute (31–300 s) phases of the adaptation (**A**,**B**). The CoBF reductions were quantified as the areas over the curve (AOCs) for each mouse and analyzed separately in the acute (**C**,**D**) and subacute (**E**,**F**) phases of adaptation. In the acute phase, no differences were observed among the experimental groups in any of the hemispheres (**C**,**D**). However, in the ipsilateral hemisphere, the TT-VDR^∆/∆^ group showed impaired compensation during the subacute phase compared to the WT and OVX-WT groups, which can be identified based on the significantly higher AOC values (**E**). In the subacute phase of the contralateral hemisphere, no significant differences were found among the experimental groups (**F**). Data are presented as mean ± SEM, *n*_WT_ = 10, *n*_VDR_^∆/∆^ = 8, *n*_OVX-WT_ = 9, *n*_OVX-VDR_^∆/∆^ = 10, *n*_TT-WT_ = 10, *n*_TT-VDR_^∆/∆^ = 9. Two-way ANOVA followed by Tukey’s post hoc test (* *p* < 0.05) was used for statistical analysis.

The temporal region is primarily supplied by the MCA [55]; thus, the most pronounced reduction in blood flow after CAO was observed here (Figure 7A). Conversely, the temporal cortex of the contralateral hemisphere, which is located the furthest from the occluded vessel, showed the least extent of CoBF decrease after CAO (Figure 7B). Figure 7A,B present the recovery patterns of the temporal region in each hemisphere. Similar to the other regions, no significant differences were observed among the groups in the acute phase in the ipsilateral hemisphere (Figure 7C). However, the TT-VDR^∆/∆^ group showed higher AOC values in the subacute phase compared to the WT and OVX-WT groups, indicating an impaired adaptational capacity in TT-VDR^∆/∆^ mice (Figure 7E). In the contralateral hemisphere, no significant differences were found among the groups in either the acute (Figure 7D) or in the subacute (Figure 7F) phases of adaptation.

## 4. Discussion

Despite the growing concern for vitamin D deficiency (VDD), its prevalence in the population is still estimated to be between 24 and 40% [60]. Notably, women, after reaching menopause, show a higher prevalence of VDD and are more susceptible to developing cardiovascular and cerebrovascular diseases [59,61]. Endocrine disorders characterized by androgen excess in women can occur at any stage of life and are not only associated with an increased risk of cardiovascular and cerebrovascular diseases but are also often accompanied by VDD [45,62]. In the present study, we examined the combined effect of hormonal imbalances (i.e., estrogen deprivation or hyperandrogenism) and disrupted vitamin D signaling on the cerebrocortical adaptation to CAO in female mice.

Rodent models have been widely used to investigate the effects of VDD, mainly through the application of vitamin-D-deficient diets or genetic modifications, particularly those of VDR [63,64]. The functional inactivation of VDR used for our experiments is a commonly employed method to investigate the consequences of disrupted vitamin D signaling [52,65]. The latter model mimics a rare human hereditary disorder called vitamin-D-dependent rickets type II, which is characterized by the unresponsiveness of VDR to vitamin D [65,66]. In our study, estrogen insufficiency characteristic of menopause was induced through surgical ovariectomy in VDR-mutant mice. The cerebrocortical blood flow changes were assessed five weeks after surgery, by which time the estrus cycle of the ovariectomized mice had ceased, confirming the successful removal of the ovaries [54,67]. To induce androgen excess, female mice received daily testosterone treatment for five weeks. Despite the serum testosterone levels of our control mice being out of the measurable range, a comparison with literature data regarding normal values in female mice revealed several-fold elevations in the androgen levels of our treated groups [68]. This demonstrated the efficacy of the five-week transdermal treatment, which managed to raise the testosterone levels between those observed in normal female and normal male mice [52,68], similar to the elevation observed in hyperandrogenic women [69]. Furthermore, body mass was significantly increased by both ovariectomy and testosterone treatment, consistent with previous findings [70,71,72]. The reason for this might be increased fat mass in both cases (e.g., due to higher food intake or decreased metabolic rate), although the underlying mechanisms are not completely understood yet [70,72,73]. These observations indicate that the models in our experimental design are suitable for investigating the consequences of estrogen insufficiency and hyperandrogenism. The effect of VDR inactivity, estrogen insufficiency and androgen excess on the major determinant factors (e.g., blood pressure, arterial blood gas tensions, oxygen saturation) of the cerebral autoregulatory capacity were also examined. In our experiments, none of the groups showed significant differences in blood pressure indicating that those factors did not impact arterial blood pressure. Notably, findings about the effects of ovariectomy, androgen excess, and especially VDD on blood pressure in rodent models are controversial depending on, for instance, the type of anesthesia and age of animals [36,37,38,60,70,74]. Moreover, like blood pressure, the arterial blood gas tensions (i.e., pCO_2_, pO_2_) also did not differ between our experimental groups; therefore, we could reject the possibility of differences in these factors influencing the CoBF alterations following CAO.

The CAO model used in this study is a well-established experimental model that mimics the pathophysiological conditions following occlusion of a major artery supplying the brain. This model enabled us to evaluate the efficiency of compensatory mechanisms, such as collateral circulation and vascular reactivity, providing valuable insights into the physiological mechanisms underlying cerebrovascular health and diseases. While the detrimental functional consequences of ablated vitamin D receptor signaling on the cerebrovascular system have been previously confirmed in male mice [42], the influence of sex steroids on the acute compensatory mechanisms following CAO remains uncertain. By examining the cerebrocortical adaptation to CAO in female mice with hormonal imbalances and disrupted vitamin D signaling, we could gain a better understanding of how these factors interact and influence the cerebrovascular response to ischemic conditions. In this study, we aimed to analyze the recovery patterns of cerebrocortical blood flow in VDR-mutant females after CAO in three distinct regions: frontal, parietal, and temporal. During CAO, blood flow is disrupted through the internal carotid artery and the MCA, which primarily supplies the temporal region [55]. Compensation can occur through the large arteries of the circle of Willis, acting as the initial defense against ischemia [75]. The contralateral side also plays a significant role in compensation, as the AACA supplies the frontal and parietal regions in both hemispheres, providing additional blood supply from the unaffected hemisphere to the hypoperfused area [55,76]. Leptomeningeal collaterals between the branches of the ACA and MCA can further divert blood flow to regions with more severe hypoperfusion [75,76], improving perfusion in the temporal region at the expense of the frontal–parietal regions [55]. VDD is known to impair vascular reactivity, promote vascular inflammation, and induce endothelial dysfunction, potentially impacting cerebrovascular function [42,60]. Surprisingly, in intact female mice, the lack of vitamin D signaling alone did not alter the cerebrocortical adaptation to CAO, as no significant differences were observed between intact VDR^∆/∆^ and WT mice in any of the investigated regions. These findings strongly suggest the existence of sex dimorphism in the effects of disrupted vitamin D signaling, with more pronounced alterations observed in males [42].

To assess the capacity of MCA-to-ACA collaterals to compensate for blood loss in the temporal region [42], collateral morphology was evaluated. The infarct volume following MCA occlusion is inversely associated with the number and diameter of collaterals and directly associated with the territory of the MCA [77]. The measured distance of the anastomotic line from the midline (Figure 1B) represents the border of the cerebral areas supplied by the MCA and ACA, and a shift in localization would indicate an altered proportion of their territories. Increased vascular tortuosity likely indicates disturbed hemodynamic conditions [78]. The number of collaterals decreased in both female and male VDR^∆/∆^ mice, suggesting that vitamin D signaling modulates collateral development in both sexes [79]. However, the sex difference in tortuosity and localization still implies a sex-dependent influence on the consequences of VDD [42]. Vitamin D regulates genes involved in cell proliferation, differentiation, and migration, while sex steroids can also influence these processes [1,60,80,81]. Moreover, vascular endothelial growth factor (VEGF) is a key mediator in angiogenesis [82,83,84], and both vitamin D and estrogen have been reported to upregulate the expression of VEGF and its receptors [85,86]. Overall, the less negative impact of non-functioning VDR on collateral development may contribute to more efficient compensation through anastomoses to the temporal cortex after CAO in VDR^∆/∆^ females compared to males. The limitation of the current method is that the morphology of collaterals cannot be examined in the same mouse before and after the five-week hormonal impact (i.e., ovariectomy, testosterone treatment). However, we assume that this short-term estrogen deficiency or androgen excess would not cause any alterations in the observed parameters in young, adult mice without any stimulation to angiogenesis (e.g., chronic ischemia, tumor growth) [87].

Estrogen can enhance NO-dependent vasodilation by increasing NO production through the stimulation of endothelial NO synthase expression and activity [49]. Moreover, estrogen increases the synthesis of the vasodilator prostacyclin [50]. Vitamin D can also prevent endothelial dysfunction by increasing the bioavailability of NO, reducing oxidative stress and proinflammatory cytokines, and regulating prostaglandins [60,88]. Additionally, vitamin D and estrogen have been reported to upregulate each other’s receptors [89,90], suggesting a possible interaction between them. Considering the vasoprotective role of estrogen [49], it is plausible that estrogen prevents the deleterious impact of lacking vitamin D signaling in intact females. Therefore, we hypothesized that the vasoprotective effects of estrogen might diminish after ovariectomy. In support of this hypothesis, previous studies have shown that cerebrovascular circulation may be compromised after ovariectomy [91,92,93]. For example, cerebral arteries in ovariectomized mice have been found to be more constricted with less NO-mediated dilation, while this effect was reversed by estrogen replacement [91]. Moreover, prostanoid-mediated contractions were predominant in ovariectomized rat cerebral arteries, whereas vasodilator prostanoids became preeminent after estrogen replacement [92]. Estrogen’s neuroprotective effect has also been suggested, as ovariectomized females exhibit larger infarct volumes [93]. Surprisingly, in our study, ovariectomy did not impair the compensatory mechanisms after CAO, as no differences were found in the adaptational capacity between intact (VDR^∆/∆^, WT) and ovariectomized mice (OVX-VDR^∆/∆^, OVX-WT). However, it is important to note that the mice in our study were young and only experienced estrogen deprivation for five weeks. Additionally, estrogens might have a more significant impact on the chronic outcome of ischemia rather than the acute blood flow changes after an occlusion.

While the effect of androgens on cerebrovascular function has been well described in males, its impact on females remains to be elucidated [94]. People who experience gender dysphoria often pursue hormone therapy for gender affirmation, including transgender men who undergo testosterone treatment as part of their gender transition [95]. Testosterone treatment in transgender men leads to masculinizing effects (e.g., changes in body composition, voice deepening, and facial and body hair growth); however, it is important to emphasize that testosterone treatment may also carry certain health considerations and potentially severe side effects [96]. One area of concern is the impact of testosterone on cardiovascular health. Androgens enhance the production of the potent vasoconstrictor thromboxane A2, stimulate inflammatory responses, and suppress endothelium-derived hyperpolarizing factors, leading to increased vascular tone [50]. Testosterone can also affect lipid metabolism, leading to alterations in lipid profiles, including increases in total cholesterol, LDL cholesterol, and triglyceride levels [96]. These changes, combined with other factors such as insulin resistance, could potentially contribute to an increased risk of cardiovascular diseases. In a previous study, cerebral arteries in vitamin-D-deficient female rats displayed inward remodeling and altered reactivity, but only when hyperandrogenism was present concurrently [37]. Thus, androgen excess in females may exacerbate the cerebrovascular consequences of VDD. Consistent with this notion, our in vivo experiments revealed that the cerebrocortical adaptation of TT-VDR^∆/∆^ mice was significantly worsened compared to the other experimental groups, as they experienced prolonged hypoperfusion during the subacute phase of adaptation in all ipsilateral regions (i.e., temporal, frontal, parietal). The temporal region of TT-VDR^∆/∆^ mice exhibited the most sustained hypoperfusion, but the frontal and parietal regions were also affected. The draining effect through leptomeningeal collaterals may contribute to this phenomenon, as they attempt to supply the more ischemic temporal area at the expense of the frontal–parietal regions [55]. It has also been reported that both pial collaterals and the large vessels of the Willis circle must undergo vasodilation to improve perfusion [55]. Since both VDD and androgen excess compromise endothelial function and vascular reactivity [36,37,38], their combined effect may be potent enough to impair compensatory mechanisms beyond what is observed in intact VDR^∆/∆^ and ovariectomized mice (i.e., OVX-VDR^∆/∆^, OVX-WT). Therefore, our results suggest that the deleterious cerebrovascular impact of disrupted vitamin D signaling may be exacerbated in females when it is combined with hyperandrogenism. Women with PCOS, also characterized by androgen excess, develop symptoms such as hypertension, endothelial dysfunction, increased arterial stiffness, and chronic inflammation, which contribute significantly to their higher risk of ischemic stroke [44]. According to randomized control studies, VDD has been associated with exacerbated PCOS symptoms, which can be improved by high-dose vitamin D supplementation [97,98], emphasizing the importance of vitamin D in the clinical manifestation of PCOS. The present study demonstrates the functional impairment of cerebrovascular circulation that develops due to the lack of vitamin D signaling when combined with hyperandrogenism, suggesting that their simultaneous presence might even directly aggravate the outcome of cerebrovascular diseases in the brain, such as ischemic stroke.

## 5. Conclusions

In conclusion, the findings of the present study highlight the interplay between sex steroids and vitamin D in their effect on cerebrocortical circulation. Functional inactivation of VDR itself did not impair cerebrocortical adaptation to CAO in intact females, possibly due to the less severe morphological alterations in leptomeningeal collaterals. Surprisingly, unlike hyperandrogenism, ovariectomy did not aggravate the effect of disrupted VDR signaling on cerebrovascular adaptation. Our results suggest that the lack of vitamin D signaling combined with hyperandrogenism impairs the acute compensation after CAO, as short-term chronic androgen exposure with VDR inactivity resulted in prolonged hypoperfusion in all regions of the ipsilateral cortex. These findings imply a vasoregulatory dysfunction that may contribute to the increased risk of ischemic stroke when vitamin D deficiency and hyperandrogenism coexist. Therefore, our results underscore the need for well-designed epidemiological and clinical studies to investigate the prevention strategies and therapeutical interventions for hyperandrogenism and vitamin-D-associated disorders, particularly in young females at high risk of sex hormonal imbalances, to mitigate the cerebrovascular consequences related to these conditions.

## Figures and Tables

**Table 1:** Experimental design in the in vivo cerebrocortical blood flow measurements.

Experimental Group	WT	VDR^∆/∆^	OVX-WT	OVX-VDR^∆/∆^	TT-WT	TT-VDR^∆/∆^
Vitamin D Signaling	+	−	+	−	+	−
Ovariectomy	−	−	+	+	−	−
Testosterone Treatment	−	−	−	−	+	+

The in vivo cerebrocortical blood flow measurements were performed on female mice carrying functionally inactive vitamin D receptors (VDR^∆/∆^) and wild-type (WT) littermates. The mice were assigned to six experimental groups: intact controls (VDR^∆/∆^, WT), ovariectomized (OVX-VDR^∆/∆^, OVX-WT), and testosterone-treated (TT-VDR^∆/∆^, TT-WT) groups. The ovaries were removed five weeks before the in vivo cerebrocortical blood flow measurements, and similarly, testosterone-treated mice received transdermal treatment for five weeks before them.

**Table 2:** Anatomical traits.

Parameter	WT	VDR^∆/∆^	OVX-WT	OVX-VDR^∆/∆^	TT-WT	TT-VDR^∆/∆^
Body Weight (g)	22.10 ± 0.18	20.88 ± 0.44	23.56 ± 0.42	21.82 ± 0.30	25.34 ± 1.40 *^,^**^,†,#^	22.22 ± 0.82
Heart Weight (g)	0.13 (0.12–0.14)	0.12 (0.12–0.14)	0.13 (0.12–0.13)	0.12 (0.11–0.12)	0.13 (0.12–0.14)	0.12 (0.11–0.12)
Tibial Length (cm)	1.66 ± 0.03	1.59 ± 0.04 **^,†^	1.76 ± 0.03	1.67 ± 0.03	1.75 ± 0.03	1.68 ± 0.03
Brain Weight (g)	0.442 ± 0.005	0.438 ± 0.003	0.441 ± 0.008	0.442 ± 0.009	0.451 ± 0.006	0.440 ± 0.006
Heart Weight/Body Weight (%)	0.58 ± 0.02	0.60 ± 0.03	0.53 ± 0.02	0.53 ± 0.01	0.55 ± 0.05	0.58 ± 0.03

TT-WT group had significantly higher body weight compared to intact WT (* *p* < 0.05) and VDR^∆/∆^ (** *p* < 0.01), OVX-VDR^∆/∆^ (^†^ *p* < 0.05), and TT-VDR^∆/∆^ (^#^ *p* < 0.05) females. VDR^∆/∆^ females had significantly shorter tibial length compared to OVX-WT (** *p* < 0.01) and TT-WT (^†^ *p* < 0.05) groups. None of the other parameters were different between the experimental groups. Data are presented as mean ± SEM or median and interquartile range, *n* = 10 in all groups, two-way ANOVA followed by Tukey’s post hoc test was used for statistical analysis.

**Table 3:** Mean arterial blood pressure (MABP) values during the in vivo cerebrocortical blood flow measurements.

MABP (mmHg)	WT	VDR^∆/∆^	OVX-WT	OVX-VDR^∆/∆^	TT-WT	TT-VDR^∆/∆^
Baseline	79.38 ± 3.11	76.12 ± 2.30	75.72 ± 3.34	70.54 ± 2.23	75.91 ± 2.07	75.72 ± 2.70
0–60 s	83.17 ± 3.36	80.38 ± 2.72	81.21 ± 3.36	74.91 ± 1.63	83.04 ± 1.95	82.51 ± 2.90
61–120 s	82.72 ± 3.62	79.02 ± 2.18	80.53 ± 3.00	74.94 ± 2.34	83.34 ± 2.11	80.62 ± 3.28
121–180 s	83.08 ± 3.55	77.11 ± 2.58	79.16 ± 2.98	71.59 ± 2.18	81.97 ± 2.21	78.49 ± 2.80
181–240 s	83.09 ± 3.53	76.87 ± 2.56	78.88 ± 3.11	71.28 ± 2.20	82.03 ± 2.28	78.72 ± 3.18
241–300 s	83.72 ± 3.56	76.11 ± 2.53	78.37 ± 3.04	71.57 ± 2.10	81.60 ± 2.07	79.21 ± 3.50

Baseline MABP was defined as the one-minute-long recording before carotid artery occlusion (CAO). CAO was performed at time point zero, and MABP was monitored for five minutes thereafter. The average one-minute MABP values were calculated for each mouse in the experimental groups and then analyzed statistically. The blood pressure values of all groups were in the physiological range above the lower limit of cerebral autoregulation throughout the experiment. None of the experimental groups’ blood pressures differed from the others. Data are presented as mean ± SEM, *n*_WT_ = 10, *n*_VDR_^∆/∆^ = 8, *n*_OVX-WT_ = 9, *n*_OVX-VDR_^∆/∆^ = 10, *n*_TT-WT_ = 10, *n*_TT-VDR_^∆/∆^ = 9. Two-way ANOVA followed by Tukey’s post hoc test was used for statistical analysis.

**Table 4:** Arterial blood gas, acid–base parameters, and plasma ion concentrations.

Parameter	WT	VDR^∆/∆^	OVX-WT	OVX-VDR^∆/∆^	TT-WT	TT-VDR^∆/∆^
pH	7.29 ± 0.02	7.27 ± 0.01	7.30 ± 0.01	7.28 ± 0.02	7.26 ± 0.02	7.27 ± 0.02
pCO_2_ (mmHg)	40.35 (35.30–47.03)	38.20 (35.28–47.38)	45.00 (36.65–46.90)	35.60 (31.43–45.93)	48.85 (31.70–50.85)	43.60 (38.60–45.90)
pO_2_ (mmHg)	96.50 ± 3.65	93.00 ± 4.56	94.22 ± 2.41	99.00 ± 4.81	93.4 ± 3.35	98.44 ± 3.36
Hematocrit (%)	42.10 ± 1.14	41.0 ± 1.09	39.89 ± 0.98	39.70 ± 0.60	39.30 ± 0.63	38.44 ± 0.58
cNa⁺ (mmol/L)	155.60 ± 1.19	156.10 ± 1.04	157.90 ± 1.20	157.90 ± 1.20	156.50 ± 0.82	154.80 ± 0.76
cK⁺ (mmol/L)	4.33 ± 0.13	4.35 ± 0.12	4.47 ± 0.06	4.29 ± 0.14	4.27 ± 0.10	4.34 ± 0.15
cCa^2^⁺ (mmol/L)	1.28 ± 0.02	1.22 ± 0.02	1.27 ± 0.03	1.25 ± 0.02	1.29 ± 0.01	1.27 ± 0.02
cCl¯ (mmol/L)	116.20 ± 1.87	115.80 ± 0.94	116.00 ± 1.23	115.40 ± 1.51	115.20 ± 0.92	115.10 ± 0.63
cHCO^3¯^ (mmol/L)	18.83 ± 0.82	17.90 ± 0.76	19.09 ± 0.96	18.22 ± 0.73	19.07 ± 0.28	18.84 ± 0.72
O_2_ saturation (%)	97.28 ± 0.51	96.20 ± 0.68	97.33 ± 0.34	97.25 ± 0.50	96.04 ± 0.62	97.17 ± 0.38

None of the parameters were different among the experimental groups. Data are presented as mean ± SEM or median and interquartile range, *n*_WT_ = 10, *n*_VDR_^∆/∆^ = 8, *n*_OVX-WT_ = 9, *n*_OVX-VDR_^∆/∆^ = 10, *n*_TT-WT_ = 10, *n*_TT-VDR_^∆/∆^ = 9. Two-way ANOVA followed by Tukey’s post hoc test was used for statistical analysis.

## Data Availability

The datasets recorded and analyzed in the current study are not publicly available due to the extensive mass of data, but the data are available from the corresponding authors upon reasonable request.
